# Decisional conflict of physicians during the decision-making process for a simulated advanced-stage cancer patient: an international longitudinal study with German and Belgian physicians

**DOI:** 10.1186/s12885-018-5071-5

**Published:** 2018-11-23

**Authors:** Catharina Schoenfeld, Yves Libert, Heribert Sattel, Delphine Canivet, France Delevallez, Andreas Dinkel, Pascal O. Berberat, Alexander Wuensch, Darius Razavi

**Affiliations:** 1 TUM Medical Education Center, TUM School of Medicine, Klinikum rechts der Isar, Technical University of Munich, Ismaninger Straße 22, 81675 Munich, Germany; 2 Unité de recherche en psychosomatique et en psycho-oncologie, Université Libre de Bruxelles, CP191 Avenue F.D. Roosevelt 50, 1050 Brussels, Belgium; 3 Institut Jules Bordet, Boulevard de Waterloo 121-125, 1000 Brussels, Belgium; 4 Department of Psychosomatic Medicine and Psychotherapy, Klinikum rechts der Isar, Technical University of Munich, Langerstraße 3, 81675 Munich, Germany; 5 Clinic of Psychosomatic Medicine and Psychotherapy, Medical Center – University of Freiburg, Faculty of Medicine, University of Freiburg, Hauptstraße 5a, 79104 Freiburg, Germany; 6 Psychosocial Cancer Counselling Center [Psychosoziale Krebsberatungsstelle], Comprehensive Cancer Center Freiburg [CCCF], Hauptstr. 5a, 79104 Freiburg, Germany

**Keywords:** Uncertainty, Decisional conflict, Decision making, Oncology, Intercultural

## Abstract

**Background:**

Decision making with advanced cancer patients is often associated with decisional conflict regarding treatment outcomes. This longitudinal multicenter study investigated German physicians’ course of decisional conflict during the decision-making process for a Simulated advanced-stage cancer Patient (SP). Results were compared to a matched sample of Belgian physicians.

**Methods:**

German physicians’ (*n* = 30) decisional conflict was assessed with the Decisional Conflict Scale (DCS) at baseline (t1) and after the four steps of a decision-making process: after reviewing the SP chart (t2), after viewing an assessment video interview with the SP (t3), after reviewing the team recommendations (t4), and after conducting the patient-physician decision-making interview (t5). The results were compared to those of a Belgian matched sample (*n* = 30).

**Results:**

Decisional conflict of German physicians decreased during the Decision-Making process (M = 53.5, SD = 11.6 at t2 to M = 37.8, SD = 9.6 at t5, *p* < 0.001). This was similar to the pattern in the Belgian sample (M = 53.5, SD = 12.5 at t2 to M = 34.1, SD = 10.9 at t5, *p* < 0.001). There was no significant difference between the two groups for Decisional conflict end scores (*p* = 0.171). At the end of the Decision-making process, in both groups, still 43.3% of the physicians among each group (*n* = 13) reported a high Decisional Conflict (DCS > 37.5).

**Conclusions:**

Physicians’ decisional conflict decreases during the decision-making process for an advanced cancer SP, though it remains at a high level. Culture, language and different health care systems have no influence on this process. The results emphasize the influence of psychosocial factors. We conclude that this issue should be considered more intensively in future research and in clinical care.

**Electronic supplementary material:**

The online version of this article (10.1186/s12885-018-5071-5) contains supplementary material, which is available to authorized users.

## Background

Physicians face a high amount of uncertainty when making treatment decisions in cancer care, despite the best possible protocols and most profound guidelines [1]. This uncertainty is further increased by uncertain tumour development, uncertain treatment outcomes and uncertain psychological and behavioural reactions of the patient, e.g. emotional distress or compliance with treatment. In decision-making encounters with advanced stage cancer patients, physicians may experience higher uncertainty regarding treatment outcomes as scientific evidence about the best treatment to choose is limited [2, 3]. This uncertainty may lead physicians to perceive a decisional conflict [4–6]. Decisional conflict [7] has been defined as ‘a state of uncertainty about which course of action to take when choice among competing actions involves risk, loss, regret or challenge to personal life values for oneself or for someone else [7]. Physicians decisional conflict [8, 9] could result in decreased physicians’ well-being and poor work satisfaction [10], poor decision-making process that could impair patients’ compliance with treatments [11] and the quality of cancer care [12]. A better understanding of the course of physicians’ decision-making conflict during a decision-making process involving uncertainties will allow proposing methods for improving the care of patients with advanced cancer.

Typically, decision-making process in cancer care involves four steps: first, the physician must review the patient chart regarding past and current medical, psychological and social available information. Second, a first physician-patient assessment interview should be conducted to evaluate the current state of the cancer disease as well as the patient’s wishes and expectations. For a high standard in cancer treatment, multidisciplinary rounds have been introduced to discuss all aspects of the current situation from the viewpoint of different disciplines in order to suggest the best treatment recommendations [1, 13, 14]. Thus, the third step is the team recommendations that should be reviewed by the physicians before meeting the patient again to make the decision. Finally, as a fourth step, a second physician-patient decision-making interview should be conducted where further treatment is decided [1]. Despite all standards, recommendations and professional decisional support, decision-making may remain difficult and complex.

Furthermore, little is known about potential differences in countries regarding the physicians’ decisional conflict during a decision-making process. In communication research, physicians from different countries have been found to vary in the way they conduct consultations [15, 16] and make recommendations [17]. A study of European general practitioners showed that Belgian and German physicians seem to differ in the focus of psychosocial and biomedical issues. German physicians’ emphases on biomedical issues and Belgian physicians more on psychosocial aspects [15]. Other studies on differences between physicians from different countries have shown similar findings, where slight cultural differences can be found, but the overall difficulties physicians face and the physicians’ qualities rated as important by patients were similar across countries [11, 12, 15, 17]. However, research into this matter is scarce and no previous study assessed the influence of culture, language and different health care systems on the physicians’ course of decisional conflict during the decision-making process for cancer patients.

It should be recalled that physicians’ communication may vary according to patients’ preferences, their disease status and communication behaviours. The potential treatment decisions can also vary widely. The use of standardized simulated patient has been recommended to reduce these variabilities [18–20]. This study simulated a decision-making process common in hospital cancer care. This includes the described four steps of the decision-making process.

To our knowledge, this is the first study to assess and generate hypotheses about the course of physicians’ Decisional Conflict during the decision-making process for a simulated advanced-stage cancer patient (SP). Their Decisional Conflict was assessed using the Decisional Conflict Scale (DCS). The primary objective of the study is to explore this physicians’ DCS course in a sample of German physicians at different time points. The secondary objective of the study is to explore differences between these results and those of a matched sample of Belgian physicians included in exactly the same assessment procedure.

## Methods

### Study design

The setup of this study was to let physicians run through a typical decision-making process [1] and observe their attitudes toward uncertainty during this task. The methods used in this study were first introduced by Libert et al. [8, 21] and described a prototypical case of high uncertainty. This case was designed with oncology specialists and integrated current guidelines.

**Fig. 1:**
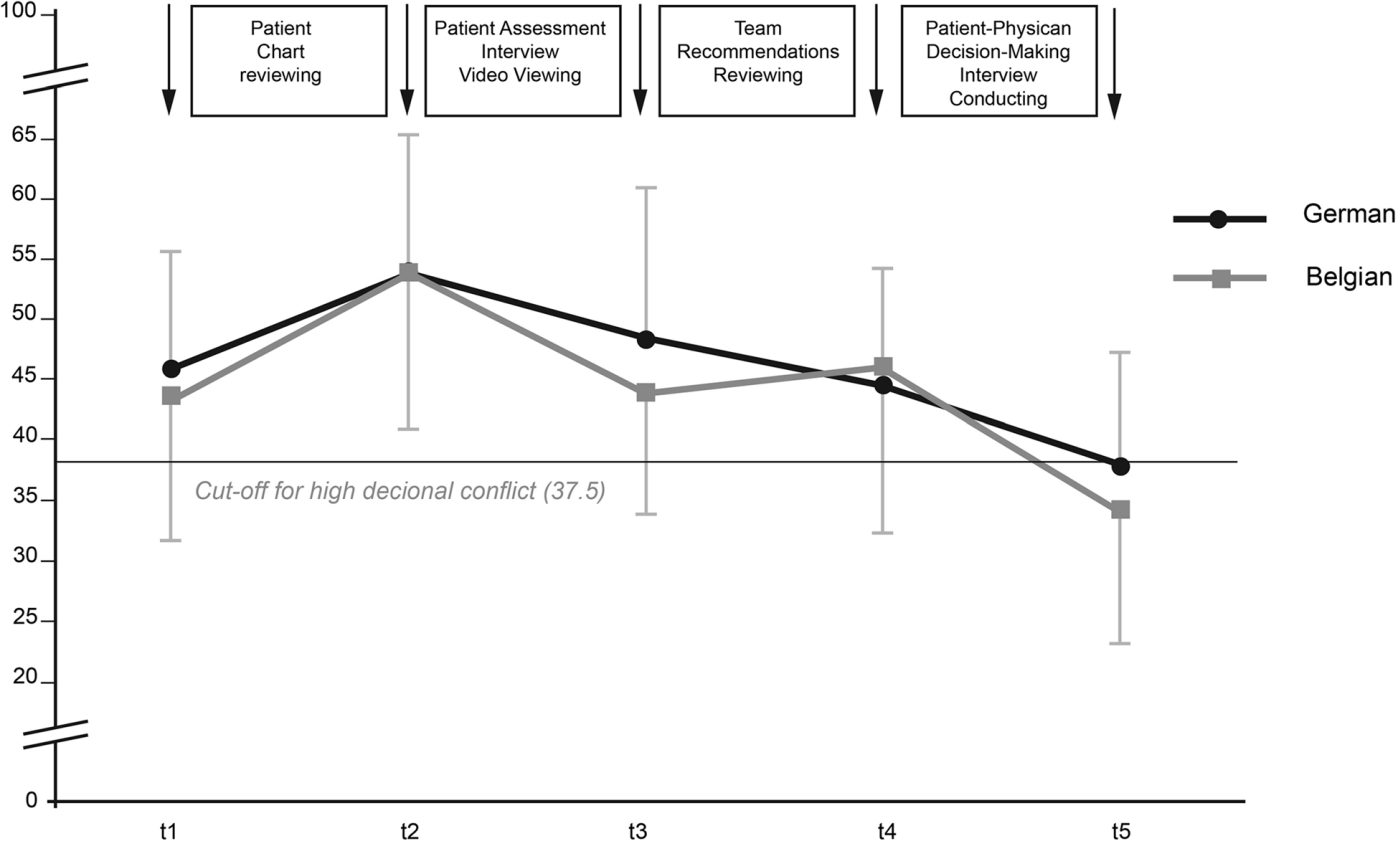
Decisional conflict. The course of German (*n* = 30) and Belgian (*n* = 30) physicians’ global Decisional Conflict during the decision-making process for a simulated advanced-stage cancer patient

This simulated case was developed in order to increase physicians’ uncertainty about medico-psycho-social components and available evidence-based treatments. Briefly described, the simulated patient chosen for this study was a 68-year-old woman with advanced cancer. She reported a second recurrence (pulmonary metastasis) of colorectal cancer that had previously been treated with surgery and chemotherapy. The decision to be made in this study was how to proceed regarding this pulmonary metastasis. Physicians had approximately 21 days to complete the task, as this time span simulates decision-making in everyday clinical life (Fig. 1).

At baseline (t1) physicians’ social and professional characteristics were assessed. Afterwards, the participants received access to the patients’ medical chart including information on the social and psychological aspects (t2).

One week later, the participants watched a 27-min standardized assessment interview between the patient and an oncologist (t3). This video gave the participating physicians further insight into the patients’ medical, social and psychological concerns as well as her wish to be involved in the decision-making process.

One week later, an interdisciplinary team recommendation for the further procedure in this patients’ case was sent to the physicians (t4). Two different treatment options were proposed in this document: The first choice of treatment was surgery of the lung metastasis with follow-up exams every two months, along with adjuvant chemotherapy and targeted therapy (Irinotecan and Cetuximab) should cancer progression be noted. The alternative proposed was chemotherapy and targeted therapy alone.

As performance assessment task (t5), physicians were asked to meet with the patient, who was portrayed by the patient-actress from the previously viewed video and to decide on the best treatment. There was no time limit for this encounter. The actress had received written instructions on her role, had seen the Belgian version of the video of the physician-patient-interview and had received constructive feedback after a trial run. To ensure that every physician is confronted with the same decision-making process and the same potential evolution of decisional conflict, the Standardized Patient (actress) involved in the simulated case was trained to refuse treatment recommendations systematically after information about treatment recommendation. She was instructed to choose supportive care as her final selection that would have only a moderate impact on her quality of life as her final selection. To increase the level of uncertainty, she was instructed at the very end to ask the physician whether her decision was a good decision. This patient role was designed with the help of oncologists and designed as a prototypical case with a very high amount of uncertainty on all levels.

The patient’s characteristics, medical history and the team recommendation given were designed by the medical oncology unit and the psycho-oncology clinic of the Jules Bordet Institute in Brussels, Belgium [8, 21]. All information was translated into German and checked against German clinical standards by oncologists.

### Questionnaires

All participants filled questionnaires before and after each task of the paradigm.

#### Decisional conflict scale (DCS)

The Decisional Conflict Scale (DCS) was used to assess the primary objective of this study [22]. It measures the patients’ conflict as perceived form the physician’s point of view. It was used as adapted by Légaré et al. [23]. By combining items from the original validated DCS [22] and from the validated Provider Decision Process Assessment Instrument [24], a French version of the Dyadic Decisional Conflict Scale was created. In addition, all items were modified to indicate the reference to cancer patients [8].

The questionnaire [25] consisted of two parts: In part A, physicians had to choose one among 12 treatment options that they would recommend for the patient at the time. Part B consisted of 24 items. Answers were given through a 5-point Likert scale (1 = strongly agree; 5 = strongly disagree). The minimum score was 0, meaning no decisional conflict, the maximum score was 100, indicating high decisional conflict. A cut off of 37.5 is defined as a high decisional conflict.

The DCS was assessed at five time points throughout the Decision-making process:

- t1: baseline general assessment on decisional conflict with cancer patients
- t2: specific assessment concerning the SP after reviewing her chart
- t3: specific assessment concerning the SP after viewing an assessment video interview
- t4: specific assessment concerning the SP after reviewing the team recommendations
- t5: specific assessment concerning the SP after conducting the patient-physician decision-making interview

#### Socioprofessional characteristics

Socio-professional characteristics assessed physicians’ age, gender, work experience and profession at baseline [8].

### Translation

All required documents were translated from French into German, then translated back into French by a native French speaker fluent in German who ensured that final German version captured the meaning of the original French version.

### Subjects

Inclusion criteria for participating physicians were at least one year of work experience with cancer patients and fluency in the local language German. According to a sample size calculation [26] a size of *N* = 30 for each group was suitable to detect significant changes within each group for consecutive assessment points for moderate effect sizes (d = 0.5) and between group differences comparing German and Belgian physicians with moderate to high effect size d of 0.7 (alpha = 0.05, power = 0.80, each).

Recruitment was done by AW, PB and CS asking physicians to participate and asking those to recommend their colleagues for recruitment according to the snow ball system till the number of 30 physicians was reached. All participants received a gift certificate for gratification.

Out of the Belgian study by Libert [8], in which 85 physicians were assessed, 30 physicians were chosen to match German physicians according to the following criteria: priority 1) Field of work and sex, priority 2) age, work experience as a physician and whether they were interns or consultants.

### Statistical analysis

SPSS Version 23.0 for PC (SPSS Inc., Chicago, IL, USA) was used to generate statistical analyses. Social and professional data and DCS were described using mean and standard deviation. T-tests and Chi-squared-tests were used to evaluate differences in means and frequencies when comparing nationalities at baseline and ANCOVAs - controlling for initial values of uncertainty - were applied for consecutive comparisons of the dimension in question.

Data are included as an additional file (Schoenfeld_DataRep_Uncertainty_BMCCancer.csv).

## Results

### Sample

The social and professional characteristics of 30 German physicians and their matched 30 Belgian physicians are shown in Table 1. The makeup of the cohorts is inhomogeneous between professions (oncologists vs. surgeons vs. radiation oncologists). This mirrors the real-life situation in clinical multidisciplinary teams. In summary, the two groups were mostly similar. However, fewer surgeons from Belgium participated in the study. To match physicians according to their fields of work, all Belgian surgeons were included in the study. This is the reason for differences seen in sex, field of work, age and work experience, as these characteristics could not be considered when finding matches for surgeons. Significant differences were only observed for work experience with cancer patients (*p* = 0.042): the Belgian physicians in our sample had more work experience than their German counterparts.

**Table 1:** Sociodemographic Data of German and Belgian physicians (*n* = 60)

	German (*n* = 30)			Belgian (*n* = 30)		
	n		%	n		%
Age (years)						
Mean		39			43	
SD		7			10	
Gender						
Male	*15*		50	*17*		57
Female	*15*		50	*13*		43
Field of work^a^						
Surgery	*18*		60	*13*		43
Medical oncology	*9*		30	*14*		47
Radiooncology	*3*		10	*3*		10
Work experience in oncology						
Mean (in years)		10			15	
SD		8			9	
Work place^b^						
Ward (inpatient)	*18*		60	*26*		87
Outpatient Clinic	*24*		80	*28*		93
Number of cancer patients on the ward/week^b^						
Mean		14			8	
SD		8			9	
Number of cancer patients in outpatient clinic/week^b^						
Mean		14			15	
SD		13			13	
Number of interdisciplinary meetings/month						
Mean		9			7	
SD		7			5	

^a^Field of work includes: surgery (general surgery, neurosurgery, orthopedics, oral maxillofacial surgery, ENT, urology, gynecology), internal medicine (general internal medicine, neurology)

^b^only counting cases with> 0 patients

*Physicians’ baseline characteristics*

For all other characteristics, no significant differences were observed.

### Levels and change of decisional conflict in German physicians

Throughout the decisional process, physicians reduced their decisional conflict, which is represented by the global score of the DCS questionnaire. The significant increase seen at the beginning of the task between t1 and t2 (from M = 45.73, SD = 10.10 to M = 53.58, SD = 11.61, *p* = 0.001) shows that the reviewing of the SP medical chart leads to a significant decisional conflict among most physicians regarding the best treatment to offer her. Physicians’ decisional conflict then gradually decreased significantly (t3: M = 48.31, SD = 12.71, t4: M = 44.72, SD = 9.72, t5: M = 37.85, SD = 9.65) (t2 vs. t3: *p* = 0.002, t3 vs. t4: *p* = 0.003, t4 vs. t5: *p* = 0.001). However, the mean Decisional Conflict score at the end of the process remained above the cut-off of 37.5, indicating the remaining presence of high Decisional Conflict.

### Comparison of decisional conflict between German and Belgian physicians

Belgian physicians also managed to decrease decisional conflict and the course of their decisional conflict was similar to that of German physicians. Differences were seen in the end score of decisional conflict, which was slightly lower and beneath cut-off for Belgian physicians (M = 34.16, SD = 10.90), but not significantly different from the German results and also still indicating presence of relevant Decisional Conflict. Also, the Belgian sample experienced a slight increase in decisional conflict after reviewing the interdisciplinary team recommendation, whereas the German sample gradually reduced Decisional Conflict throughout the process with no increase in between. The slight differences between the two groups were not statistically significant. However, at the end of the Decision-making process, in both groups, still 43.3% of the physicians among each group (*n* = 13) reported a high Decisional Conflict (DCS > 37.5). The course of Decisional Conflict of German and Belgian physicians over the Decision-making process is depicted in Fig. 1, the exact numbers and results of t-tests are shown in Table 2.

**Table 2:** Decisional conflict

	German (*n* = 30)		Belgian (*n* = 30)		
	Mean	SD	Mean	SD	P^a^
Decisional Conflict scale^a^					
Before reviewing the SP Chart (baseline), t1	45.7	10.1	43.5	11.8	0.444
After reviewing the SP chart, t2	53.6	11.6	53.6	12.5	0.966
After viewing an assessment video interview with the SP, t3	48.3	12.7	44.0	10.0	0.133
After reviewing the team recommendations, t4	44.7	9.7	45.9	13.7	0.739
After conducting the patient-physician decision-making interview, t5	37.8	9.6	34.2	10.9	0.174

^a^Scale: 5-point Likert-Scale (1 = strongly agree; 5 = strongly disagree); 24 Items; Range [0 = no decisional conflict, 100 = high decisional conflict; cut-off 37.5]^b^t-Test: comparisons of consecutive assessments controlled for initial value of Decisional Conflict Scale

*The course of German (n = 30) and Belgian (n = 30) physicians’ global Decisional Conflict during the decision-making process for a simulated advanced-stage cancer (SP)*

## Discussion

This longitudinal multicentre study reports the course of physicians’ Decisional Conflict during the decision-making decisional process for a simulated advanced-stage cancer patient. Physicians’ decisional conflict decreased, though it remained at a high level in the German and Belgian sample after conducting the patient-physician decision-making interview. The differences in this course between German physicians and a sample of matched Belgian physicians are not significant.

The observed effect sizes for within group comparisons ranged between d = 0.16 and d = 0.95, with the majority of them at least moderate to high. Between group comparisons resulted in lower effect sizes which did not exceed small effects of d = 0.37 and were thus unable to demonstrate significant differences between both samples, possibly due to the restricted sample size.

Culture, language and different health care systems do not seem to have an influence in the course of physicians’ decisional conflict in this decision-making process. Moreover, although a significant difference were observed for work experience with cancer patients between the two groups of recruited physicians, the course of physicians decisional conflict observed were similar. Other research into differences between physicians from different nations has shown similar findings, where slight cultural differences can be found, but the overall difficulties physicians face and the physicians’ qualities rated as important by patients were similar across countries [11, 12, 15, 17]. This study adds that, in the face of advanced cancer, the steps recommended in the literature for making a treatment decision do not fully reduce the physicians’ decisional conflict, regardless of their experience with cancer patients, their culture, their language or their health care systems.

It is important to recall that German and Belgian physicians’ level of conflict included in this study remains high despite an exhaustive medical record; despite a collaborative assessment interview in which the SP expressed her medical, psychological, and social concerns explicitly, as well as stated her desire to participate in the decision-making process; despite relevant team recommendations and despite a patient who clearly expresses her choice of treatment. It can therefore be assumed that the decision-making conflict observed at the end of a decision-making process is higher in real clinical situations that rarely present these facilitating components.

Another finding of this study was that physicians’ decisional conflict was increased by the simulated patient’s decision for best supportive care at the end of consultation. It can be assumed that high decisional conflict is related to this patient’s question as to whether her decision was a good decision but also to her treatment choice by itself. This choice can indeed put the physician in an uncomfortable state due to its opposition to the team’s recommendations and/or due to their feeling of helplessness in the face of disease progression. It can also increase the physicians’ discomfort due their lack of knowledge of the potentially positive impact of supportive care in the context of advanced diseases on the quality and quantity of life of patients [27].

Applying a standardized assessment can be seen as a limitation and strength of the study. The study limited external validity through its experimental character. Its’ strength lies in evening out variance of patient characteristics through standardization and therefore focussing solely on the decision-making process.

As physicians from different fields of oncology showed similar patterns of decisional conflict, we assume that generalization is given. But to prove this assumption, other studies are needed.

## Conclusions

Our study reveals the complexity of decision-making processes for clinicians. Physicians may be aware that uncertainty and the resulting Decisional Conflict might still be present at the end of a decision-making process. Culture, language and different health care systems do not seem to have an influence in this process.

The results emphasize the influence of psychosocial factors. We conclude that this issue should be considered more intensively in future research and in clinical care.

## Additional file

Additional file 1: Data Repository for study data. In this file all relevant data to be analysed are included. (CSV 5 kb)

## Abbreviations

DCS: Decisional conflict scale
SP: Simulated advanced-stage cancer Patient
